# Evaluation of major mite allergens from European standardized commercial extracts for in vivo diagnosis: addressing the need for precision medicine

**DOI:** 10.1186/s13601-019-0254-y

**Published:** 2019-02-25

**Authors:** Ruperto González-Pérez, Paloma Poza-Guedes, Yvelise Barrios del Pino, Víctor Matheu, Inmaculada Sánchez-Machín

**Affiliations:** 1 Allergy Department, Hospital Universitario de Canarias, 38320 Tenerife, Spain; 2 Immunology Department, Hospital Universitario de Canarias, Tenerife, Spain

**Keywords:** Allergen, Biological standardization, Skin prick test, House dust mites, *Dermatophagoides pteronyssinus*, *Blomia tropicalis*, Precision medicine

## Abstract

**Background:**

Skin prick testing is the first-line interventional method to diagnose IgE mediated allergic diseases. Methodological differences in manufacturing processes and extract standardization may lead to variations in the reagent quality and potency. The current study evaluates sixteen commercially available *Dermatophagoides pteronyssinus* and *Blomia tropicalis* extracts for allergy diagnosis from different European manufacturers regarding allergen composition and content and whether these differences could influence their biological activity.

**Methods:**

Mite-allergic subjects (n = 21) were skin-tested with the extracts and studied for immunoglobulin E reactivity. Nine extracts from *D. pteronyssinus* and seven from *B. tropicalis* were analysed for total protein content by Bradford and ELISA double sandwich was used to quantify specific antibodies for *D. pteronyssinus* and *B. tropicalis* major allergens from nine different manufacturers.

**Results:**

Mite extracts showed a 10–60 fold variation regarding the total protein content. The contents of the major allergens of *D. pteronyssinus* and *B. tropicalis* differed considerably (30–53 fold change) among the extracts. Blo t 5 was quantitatively present in < 50% of the of the *B. tropicalis* reagents and could not be clearly detected by immunoblotting in the majority of the *B. tropicalis* commercial extracts.

**Conclusions:**

Certain natural *D. pteronyssinus* and *B. tropicalis* extracts lack important allergens showing a considerable variability in composition and content. A closer collaboration among clinicians, allergen manufacturing companies and regulatory agencies to improve the quality and consistency of *D. pteronyssinus* and *B. tropicalis* extracts is warranted to achieve a more precise diagnosis and treatment of house dust mite allergy.

## Background

House dust mites (HDM) are one of the most prevalent sources of indoor allergens throughout the world [1, 2]. Mites sensitize and induce atopic disease in predisposed individuals and are an important spoiling factor in patients with allergic rhinitis, asthma and atopic dermatitis [3]. The *Dermatophagoides* genus is the most researched of all the HDMs, although species serological dominance varies geographically, suggesting specialist adaptation [4]. *Blomia tropicalis* was originally characterized as a storage mite but nowadays constitutes an emerging key allergen, especially, but not limited to the tropical and subtropical regions of the world [5].

In vivo diagnosis of mite allergy in routine clinical practice is mainly based on clinical history and skin prick tests (SPT) with commercial extracts which is nowadays considered the first-line interventional method used to diagnose IgE mediated allergic diseases for patients with respiratory symptoms [6]. Skin prick test is reproducible, minimally invasive, relatively easy when performed properly, and allows for the testing of multiple allergens at once [7]. The panel of reagents is variable and generally depends on the prevalence of local aeroallergens [8].

Meanwhile mite allergen extracts are crucial to diagnose and treat mite allergy, a major allergen is recognized by IgE antibodies of > 50% of patients allergic to the allergen source [9]. In fact, mite immunotherapy represents approximately 50% of the total volume of marketed vaccines mainly of the genus *Dermatophagoides* [10]. Nowadays, allergen standardization concentrates on the safety issue by determining the overall immunoglobulin E (IgE)-binding potency of the allergen extracts [11]. However, each manufacturer uses company-specific units which are not suitable for the comparison of different products globally [12].

In addition, immunodetection analyses of HDM extracts indicate a complex pattern of IgE binding, and since IgE responses to one allergen may induce responses to bystander antigens, collateral responses would also be expected setting the need to identify the main allergens that drive sensitisation [13]. Furthermore, it has been shown that the concentration of major allergens correlates with the biological potency and IgE reactivity of allergen extracts [14]. Casset et al. [12] showed that in almost one-third of the Italian mite-allergic subjects, negative SPTs readings were obtained with at least 1 of the mite commercial extracts tested.

Standardized allergen extracts ideally should have a batch-to-batch consistency and the skin test results comparable when the same extracts from different manufacturers are used [15]. As allergen extracts are biological mixtures containing a variety of different proteins, glycoproteins and polysaccharides, SPT results obtained with the same allergen with extracts from different manufacturers and even different batches of allergen from the same vendor, vary [16–18]. Sensitization to many sources of allergen is preferentially directed to a small number of proteins, the dominant allergens that have stimulated the immune system. The group 1 and 2 allergens of *Dermatophagoides* spp. typically account for 50–80% of the IgE binding attributed to HDM extracts and Blo t 5 from *B. tropicalis*, account for 60% of its IgE-binding activity [19].

Despite the interpretation of SPT results are critical for a correct diagnosis and precise therapeutic interventions, very scarce information is currently available about the sensitivity of commercial extracts for SPT commonly used in the diagnosis of mite allergy in Spain. The current study evaluates sixteen European commercially available mite extracts from different manufactures regarding allergen composition and content and whether differences may affect their allergenic activity.

## Methods

### Patients

We selected 21 non-consecutive patients sensitized to both *Der p.* and *B. tropicalis* with persistent moderate to severe rhinitis according to the ARIA Guidelines [20]. Skin prick test (SPT) with standardized extracts of *Der p.* and *B. tropicalis* were performed in the forearm followed by immediate reading after 15 min. Serum blood samples were obtained from all participating subjects. Pregnant and breast-feeding women were excluded. The study was approved by the local Ethical Committee of our Institution and informed consent was signed by all subjects and parents/guardians for those participants < 18 years old.

### Extracts

We compared nine different commercially available extracts—2 batches from each company—in Spain from *Der p.* (extract 1: Diater, extract 2: ALK-Abello, extract 3: Leti, extract 4: Stallergenes-Greer, extract 5: Roxall, extract 6: Inmunotek, extract 7: Probelte, extract 8: Merck and extract 9: Hal Allergy) and 7 from *B. tropicalis* (extract 1: Diater, extract 2: ALK-Abello, extract 3: Leti, extract 4: Stallergenes-Greer, extract 5: Roxall, extract 6: Inmunotek and extract 7: Probelte). Investigators in the clinical and laboratory settings were not aware of the preparation they were using at the time of the study. The extracts under investigation were checked for expiration date, simultaneously packed in masked vials and analyzed in parallel. All reagents were codified without the name of the company and randomly numbered (i.e. *Der p.* manufacture #1–9 and *B. tropicalis* manufacture #1–7).

### Protein content

The total protein concentration of the allergenic extracts was measured using the Bradford method [21]. A standard curve ranging from 2 to 10 µg/mL of bovine serum albumin were constructed. Each experiment was performed at least 3 times, and each point was tested in duplicate. The statistical analysis was performed using SPSS^®^ packet.

### Sds page

Proteins from *Der p.* and *B. tropicalis* extracts were separated by electrophoresis with use of reduced conditions and 15% polyacrylamide running gel according to Laemmli [22]. All samples of each commercial allergenic extract were loaded with equal protein content (0,5 µg/lane). A protein molecular weight marker (Bio-Rad, Hercules, CA, USA) was used as a standard. Proteins were visualized using Silver dye staining (Thermo Fisher Scientific, Waltham, USA).

### IgE: immunoblotting

The allergenic extracts were separated in 15% SDS PAGE and transferred to a PVDF membrane (Bio-Rad, Hercules, CA, USA). The immunoblotting was performed as previously described [23]. Briefly, 5 ml of pool of sera from sensitized patients was incubated with the membrane overnight at 4 °C in agitation. Afterwards, 5 ml of a 1:10,000 dilution rabbit anti-human IgE peroxidase conjugate (DAKO P0295) was used as second antibody. For the development of the reaction, ECL plus Western Blotting Detection Reagents (Perkin Elmer Life Science, Boston, USA) was used according to the manufacturer indications.

### Major allergens content: ELISA double sandwich

The major allergens Der p1, Der p 2 and Blo t 5 content of the extracts was analysed using a commercial kit according to the manufacturer’s instructions (INDOOR biotechnologies, Charlottesville, USA).

### CAP inhibition

A pool of patients’ sera characterized using ImmunoCAP ISAC (Phadia, Thermo Fisher Scientific, Waltham, USA) a specific IgE levels of *D. pteronyssinus* and *B. tropicalis* were separately incubated overnight at 4 °C with different proteins concentration (0.1–10 µg/mL) of commercial extracts of *Der p.* and *B. tropicalis*. The allergenic proteins of *D. pteronyssinus* and *B. tropicalis* present on ImmunoCAP were Der p1, Der p2, Der p10, Der p 23 while Blo t5 was displayed on ISAC. The CAP-inhibition test was carried out with a specific programme in UniCap 100 (Phadia, Thermo Fisher Scientific, Waltham, USA) towards all 16 commercial allergenic extracts of *D. pteronyssinus* and *B. tropicalis*.

## Results

### Protein content

A quantitative analysis of total proteins was performed in order to evaluate the level of heterogeneity of the extracts from *Der p.* (9) and *B. tropicalis* (7) from different manufactures. The protein concentration of *Der p.* ranged from 0.023 mg/mL of extract to 0.143 mg/mL, whereas the protein concentration of *B. tropicalis* ranged from 0.015 to 0.875 mg/mL (Table 1).

**Table 1:** Protein content (mg/mL) in commercial extracts from manufacturers 1 to 9

Manufacturer	*D. pteronyssinus*	*B. tropicalis*
1	0.105	0.875
2	0.114	0.151
3	0.236	0.029
4	0.023	0.015
5	0.143	0.453
6	0.046	0.098
7	0.098	0.164
8	0.148	–
9	0.134	–

### Major allergens content

The major allergens—Der p 1, Der p 2, and Blo t 5—content was also investigated in the individual extracts. Der p 1, Der p 2, and Blo t 5 ranged from 1.21 to 30.6 μg/mL, 0.55 to 20.49 μg/mL and 0.0 to 2.15 μg/mL, respectively (Table 2). The concentration of *Der p.* group 2 in the individual extracts was lower than group 1, except for extracts #1 and #4. In addition, the Der p 1/Der p 2 ratios differed between 0.28 and 15.62 μg/mL. Regarding *B. tropicalis*, Blo t 5 was not detected in commercial reagents from manufacturers 3, 4, 6 and 7.

**Table 2:** Major allergens content (Der p 1, Der p 2 and Blo t 5) in commercial extracts from manufacturers 1 to 9. Results are shown in μg/mL

Manufacturer	Der p 1	Der p 2	Blo t 5
1	1.21	4.32	2.15
2	11.95	9.12	0.628
3	26.25	20.49	N.D.
4	3.06	6.06	N.D.
5	30.16	1.93	0.040
6	4.64	2.23	N.D.
7	8.48	5.66	N.D.
8	4.62	2.97	–
9	3.96	0.55	–

### Allergenic profiles

**Fig. 1:**
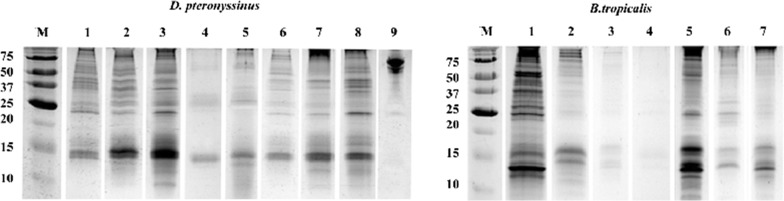
SDS PAGE of commercial extracts (*D. pteronyssinus*) from manufacturers 1 to 9

**Fig. 2:**
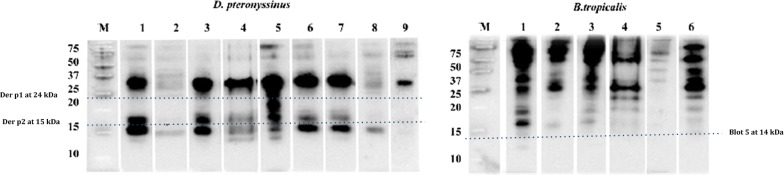
IgE Immunoblotting of commercial extracts (*D. pteronyssinus* and *B.tropicalis*) from manufacturers 1 to 9

All extracts were analysed by means of SDS-PAGE as shown in Figs. 1 and 2. The separated proteins of some extracts were at or below the detection limit of staining with silver stain. Intensities for specific components varied among extracts from different manufacturers. Moreover, some components are absent in a few *Der p.* and *B. tropicalis* extracts. Specifically, *Der p.* extracts from manufacturers 1, 2, 3, 7 and 8 showed a comparable intensity. In the extracts 4, 5, 6 and 9 bands have a lower intensity regarding detection of staining at 24 kDa (Der p 1) while extract 9 showed no detection at 15 kDa (Der p 2) nor 14 kDa (Der p 23), given that the latter was not quantified. The protein profile of *Der p.* extract 9 is apparently unique due to the presence of a prevalent component at about 73–75 kDa—*a Der f 28*-*like allergen according to the Uniprot database* [24]—without other relevant components.

*B. tropicalis* commercial extracts 1 and 5 have a similar intensity, extracts 2, 6 and 7 bands have a lower intensity regarding detection of staining at Blo t 5 (14 kDa) meanwhile this major allergen is absent in extracts 3 and 4. Blo t 21 (13 kDa) a major allergen from *B. tropicalis* was detected in extracts 1, 5, 6 and 7. Components belonging to 56 kDa (Blo t 4), were only present in the *B. tropicalis* extracts from 2 manufacturers.

Protein content and protein profile results were completed by information on allergenic properties of individual extracts including immunoblotting assays with a pool of sera from 21 patients—*14 females and 7 males, ranging from 11 to 46* *years old*—with confirmed mite allergic rhinitis, (i.e. showing a positive SPT reading to *Der p.* and *B. tropicalis*) from the Allergy outpatient clinic. The major allergens belonging to group 1 (24 kDa), group 2 (15 kDa) and Blo t 5 (14 kDa) were differently shown, only one group was detected in some cases or none of them recognized depending on the extract (Fig. 2). In fact, Blo t 5 was quantitatively not found in 4 out of the 7 *B. tropicalis* reagents (57.16%) and could not be either clearly detected by immunoblotting in 6 of them.

### Biological potency: CAP inhibition

**Fig. 3:**
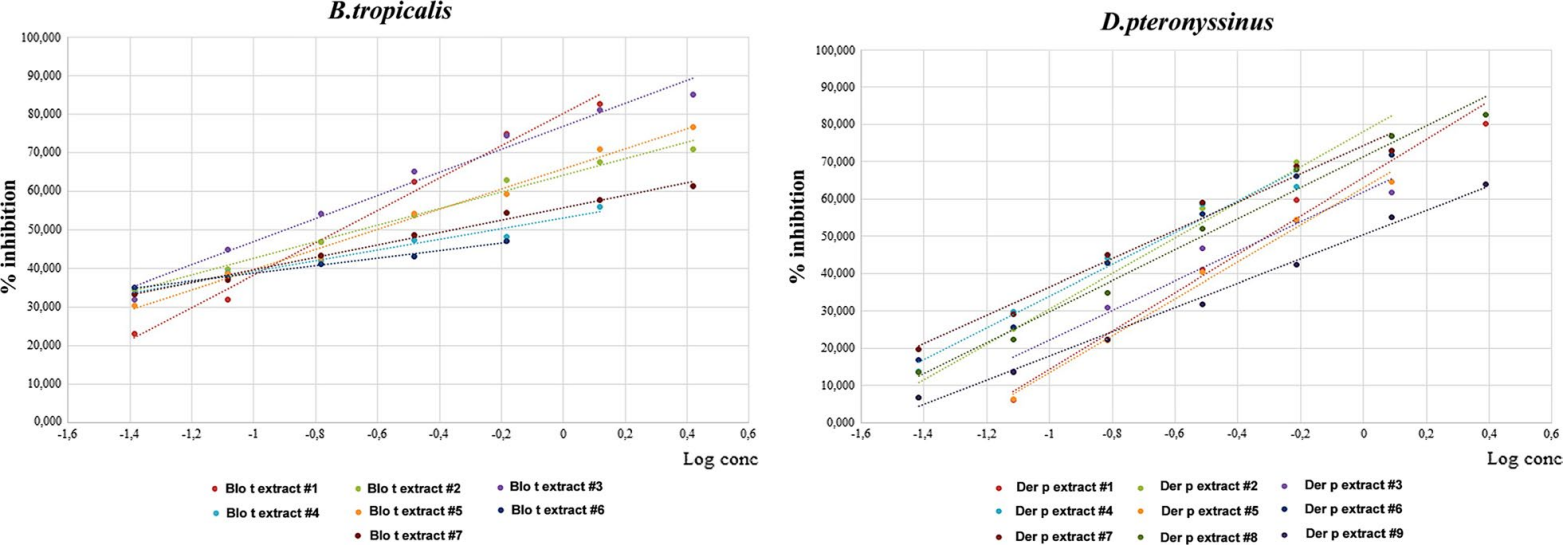
CAP inhibition of commercial extracts (*B. tropicalis* and *D. pteronyssinus*) from manufacturers 1 to 9

CAP inhibition assays (Ag50) with extracts of *Der p.* and *B. tropicalis* from different manufacturers and a pool of sera ranged from 0.228 to 0.962 μg/mL, and 0.129 to 1.398 μg/mL, respectively (Fig. 3 and Table 3).

**Table 3:** Biological potency (Ag50) of commercial extracts (*D. pteronyssinus* and *B. tropicalis*) from manufacturers 1 to 9

Manufacturer	[Ag 50] *D. pteronyssinus*	[Ag 50] *B. tropicalis*
1	0.493	0.191
2	0.255	0.222
3	0.494	0.129
4	0.235	0.586
5	0.544	0.249
6	0.265	1.398
7	0.228	0.440
8	0.305	–
9	0.962	–

## Discussion

Allergen standardization sets the basis for better and more reproducible allergen extracts. Regulatory agencies provide evidences—*Good Manufacturing Practices*—for the manufacturing and quality control of licensed allergenic extracts concerning the need for product specifications, analytical procedures, process validations, microbiological attributes, and labelling [25–27]. However, most existing regulations exempt non-standardized or “named-patient products” from the more rigorous licensing requirements in terms of safety and efficacy [28].

The variations in the biological activity among allergen extracts and manufacturers are dependent on the origin of the source material, the extraction and reconstitution process—*based on arbitrary in*-*house reference standards*—and also the clinical features of the referenced allergic subjects [29, 30]. Currently, mites are being commercially grown on a variety of food substrates, and the purity of the mite bodies in the total extract varies among the different sources and different countries [31]. In fact, it has been proven that mite-culturing diet directly effects population growth, the dynamics of allergen accumulation, the group 1/2 allergen ratio and the endotoxin contents in extracts of cultured HDM [32]. The results of our study indicate that certain natural *Der p.* and *B. tropicalis* extracts lack important allergens showing a considerable variability in the allergen composition and content. The protein content and the major allergens—Der p 1, Der p 2, and Blo t 5—found in the individual extracts was quite heterogeneous among the different manufacturers. The *Der p.* extracts showed a tenfold variation of the total protein content and the *B. tropicalis* extracts an almost 60-fold variation. The levels of the major allergens of *Der p.*, Der p 1, Der p 2, ranged from 1.21 to 30.6 μg/mL (a 30-fold variation) and 0.55–20.49 μg/mL (37-fold variation) respectively, showing a quantitative lower range compared to those commercial extracts previously published in Italy [33] (Der p 1: 9.6–36.2 μg/mL, Der p 2: 0.7–31.7 μg/mL), Austria [12] (Der p 1: 6.0–40.8 μg/mL and Der p 2: 1.7–45 μg/mL) and Korea [34].

In addition, the Der p 1/Der p 2 ratios differed considerably among extracts from 0.28 to 15.62 showing lower levels compared to previous reports from different European and Asian manufacturers [35]. Interestingly, the concentration of Der p 1 in the individual extracts was greater than Der p 2, except for extracts #1 and #4. This variation in ratios might be related to the source material—*mite feces and/or of mite bodies*-, used for extraction from each manufacturer [36].

Although more than 23 *D. pteronyssinus* allergens have been characterized [37], several studies have indicated that group 1 and 2 allergens represent the clinically most important mite allergens, which are in fact the major allergens in our population [38, 39]. It has been shown that a combination of both *D. pteronyssinus.* Allergens allow diagnosis of more than 95% of patients with HDM allergy [40]. In contrast, Casset et al. [12] confirmed no correlation between Der p 1 or Der p 2 concentrations in the extracts and the intensity of the skin response, indicating the relevance of *minor* allergens such as Der p 5, 7, 10 and 21 for the biological response.

In the present study, the major allergen from *B. tropicalis* Blo t 5 ranged from 0.0 to 2.15 μg/mL. It is remarkable that Blo t 5 was only detected in 3 out of the 7 commercially available extracts—*a 53*-*fold variation*—from *B. tropicalis*. Interestingly, relevant components belonging to Blot4 (56 kDa)—*a locally serodominant regional allergen in China* [41] *and Spain* [42]—were only detected in the *B. tropicalis* extracts from manufacturers 1 and 5, therefore a precise diagnosis may not be achieved with all the tested reagents in specific populations.

In this regard, Cardona et al. described the kinetics of released allergens during the growth cycle of *B. tropicalis* and *B. kulagini* confirming that mite cultures during the maximum growth contain the largest number of allergenic components as well as the highest Blo t 5 concentration [43].

Since both the protein and the major allergens contents may not necessarily have an impact at the appropriateness of the extracts for diagnosis, in terms of their potential IgE binding, CAP inhibition assays with 16 extracts of *Der p.* and *B. tropicalis* and a pool of sera of moderate-severe rhinitic subjects were carried out to confirm variable patterns of IgE recognition—*specially for B. tropicalis*—depending on the evaluated extract (Fig. 2).

The present results are similar to previous studies that pointed out marked differences from different manufacturers confirming that SPT extracts are so heterogeneous that a few of them are incapable of showing a positive response in some patients, stating that absence of important components can affect the diagnosis of HDMs allergy [33, 44, 45]. The current data display that no clear-cut progress have been made yet concerning this issue.

Although the commercial extracts for *Der p.* and *B. tropicalis* included in the study claimed to be biologically standardized to each in-house reference standards, wide differences in terms of allergen composition and content were found. Despite SPT has been previously described as reproducible [7], the results of this study are in not in line with this remark regarding the accuracy of HDM allergy diagnosis. However, it has been shown that standardized extracts are not necessarily more potent than conventional extracts based on weight per volume (w/v) or protein nitrogen units [46].

Mass spectrometry (MS) has been used as a tool to identify novel allergens or to prove identity and enhance the quality control of recombinant allergens [47]. Spiric et al. [48] suggest that a MS technique known as multiple reaction-monitoring (MRM) may complement the overall potency measurements or quantitation of allergenic proteins by ELISA to inform practitioners of compositional differences among HDM reagents, and of variations in composition among lots from each manufacturer.

Our investigation has some limitations, the possibility of degradation resulting from storage condition and duration of storage may be responsible for some of the variation among extracts, even though the reagents were properly stored and evaluated within the documented expiration date. Noteworthy, Jeong et al. [49] showed that despite almost 40% of total protein was degraded, more than 90% of IgE reactivity was preserved during the first 2 months of storage when reconstituted in 50% glycerol and about 80% of Der p 1 content was still preserved in the extracts. Although quantification of relevant allergens for *Der p.* (Der p 5, 7, 10, 21 and 23) and *B. tropicalis* (Blo t 4 and 21) was not performed, stain intensities for specific components were variable or even absent among extracts from different manufacturers in a few *Der p.* (extracts #4, 5, 6 and 9) and *B. tropicalis* (extracts #2, 3, 4, 6 and 7).

The probability of a given sensitization to be clinically relevant depends on the type of allergen and country where the patient lives [50]. The findings of the current study provide relevant information for the daily allergy practice in search of a more accurate diagnosis. Although, variation in therapeutic extracts [51] have been elegantly demonstrated before, no studies comparing commercially available *D. pteronyssinus* and *B. tropicalis* SPT reagents has been previously conducted in Spain. Indeed, we could not either find in the literature review any investigation regarding to the biological activity and quantification of major allergens in a set of different commercial extracts from *B. tropicalis*.

In our view, greater efforts should be made to implement a closer cooperation between allergen manufacturing companies and regulatory agencies to improve the quality and consistency of mite extracts not only in terms of comparing in-house reference standards but also among different companies. In the other hand, clinicians should bear in mind the limitations of currently available mite diagnostic extracts given that the types and levels of mite allergens that individuals are exposed to may differ to those contained in the commercially available extracts for SPT. Additionally, this study also serves as an inspiring attempt to develop “*a la carte*” instead of “*one*-*size fits all*” diagnostic extracts that can truly reflect the allergenic profile specificities of each population aiming for a better personalized medicine.

## Abbreviations

HDM: house dust mite
SPT: skin prick test
*D. pteronyssinus*: *Dermatophagoides pteronyssinus*
*B. tropicalis*: *Blomia tropicalis*
